# Occult Cranial Cervical Dislocation: A Case Report and Brief Literature Review

**DOI:** 10.1155/2016/4930285

**Published:** 2016-06-05

**Authors:** Joshua B. Shatsky, Timothy B. Alton, Carlo Bellabarba, Richard J. Bransford

**Affiliations:** Department of Orthopedics and Sports Medicine, Harborview Medical Center, 325 Ninth Avenue Seattle, WA 98104, USA

## Abstract

*Study Design*. Retrospective case report and review.* Objective*. Cranial cervical dislocation (CCD) is commonly a devastating injury. Delay in diagnosis has been found to lead to worse outcomes. Our purpose is to describe a rare case of occult cranial cervical dislocation (CCD) and use it to highlight key clinical and radiographic findings to ensure expedited diagnosis and proper management avoiding delays and subsequent neurologic deterioration.* Method*. Case report with literature review.* Results*. We describe a unique case of occult cranial cervical dislocation where initial imaging of the cervical spine failed to illustrate displacement of the occipital-cervical (O-C1) articulation or C1-C2 articulation. Careful evaluation of subtle radiographic clues suggested a more severe injury than initial review. Additional imaging was obtained due to these subtle clues confirming true cranial cervical dislocation allowing subsequent treatment with no neurologic sequelae.* Conclusion*. A high index of suspicion of CCD may prevent injury in select patients who present without gross cord compromise. Careful consideration of associated fractures, soft tissue injuries, and mechanism of injury are essential clues to the correct diagnosis and management of injuries to the craniocervical junction (CCJ).

## 1. Introduction

Injury to the craniocervical junction can be devastating. Delays in diagnosis or failure to appreciate this injury lead to neurologic deterioration and poor outcomes. Much of the initial understanding of this injury was from postmortem evaluation of traumatized patients [1]. These reports found significant disruption to the ligamentous structures of the craniocervical junction (CCJ) [1] and many believed these injuries were not survivable.

The stability of the CCJ is provided by boney articulation and both intrinsic and extrinsic ligamentous connections [2]. There are multiple osseous contact points between the occiput, atlas, and C2, which allow rotation and provide a structural foundation [2]. The primary stabilizing structures however are intrinsic ligaments and capsules [3]. From ventral to dorsal these are the alar ligament (oblique fibers connect the dens to occipital condyles), cruciate ligament (connects posterior dens to anterior atlas arch, with strong horizontal transverse atlantal ligament, and vertical fibers connecting the posterior atlas to the foramen magnum), and tectorial membrane (cranial extension of the posterior longitudinal ligament connecting posterior C2 to the anterior foramen magnum).

As the quality of field response evaluation, treatment and immobilization has improved, reports of patients surviving craniocervical dissociation (CCD) emerged [4]. Chaput et al. reported 16 cases of CCD after high energy trauma with 6 deaths from upper cervical cord transection [5]. In 2006, Bellabarba et al. described 17 consecutive CCD survivors [6]. They found 16/17 had abnormal Harris measurements on screening plain X-rays and 38% of patients were not diagnosed until after the onset of neurologic deterioration [6]. One patient had normal initial cervical spine radiographic measurement and reduced osseous articulations, consistent with dislocation and recoil reduction, highlighting the difficulty in identifying these injuries. Not all patients however have abnormal measurements as described by Harris measurements and these patients can be more challenging in which to make the diagnosis [6]. As the awareness of this injury pattern increases so does the ability of the provider to make an accurate diagnosis on initial imaging studies [7].

We present a case of occult cranial cervical dislocation, which could have easily been missed without careful consideration of associated fractures, soft tissue clues, and mechanism. Because this injury was recognized, devastating neurological and life-threatening consequences were avoided.

## 2. Case

**Figure 1:**
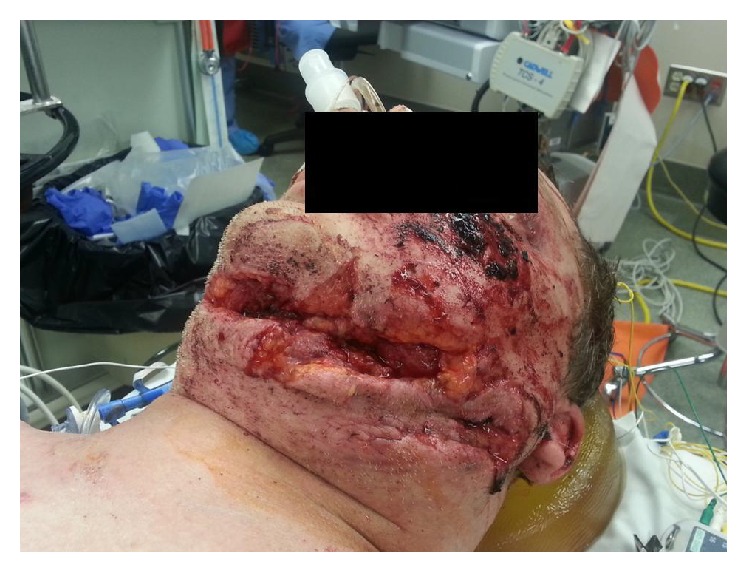
Clinical photograph illustrating severe facial trauma and soft tissue injury.

A previously healthy 49-year-old male was the unrestrained, ejected driver of a single car rollover accident. He was found unresponsive but hemodynamically stable and was taken to the local rural hospital in a Philadelphia collar where he was initially awake and following commands and found to be neurologically intact. He became combative and was nasally intubated for airway protection. He was noted to have severe facial trauma and complex neck lacerations (Figure 1). Given his mechanism of injury and significant soft tissue trauma and deteriorating clinical picture, a whole body computed tomography (CT) scan was performed. Imaging showed multiple facial and rib fractures, a Type II odontoid fracture and thoracolumbar spine injuries. The decision was made to transfer the patient to a level 1 trauma center for further management.

**Figure 2:**
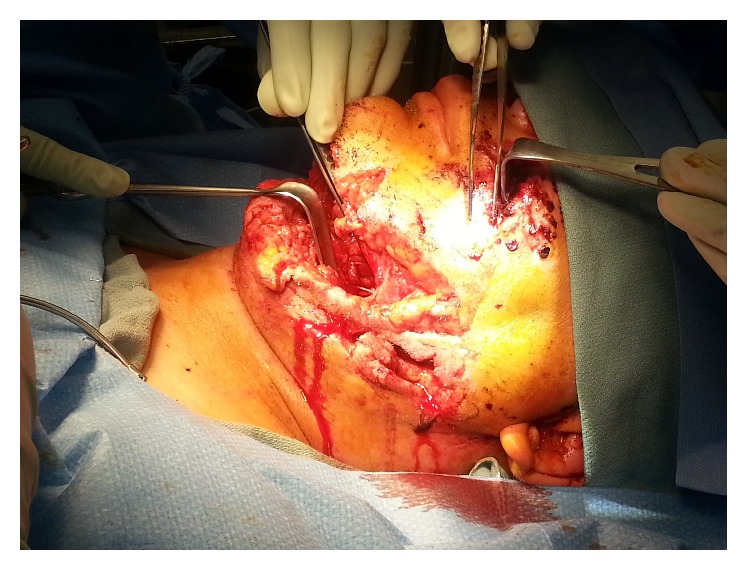
Intraoperative photo highlighting extent of facial trauma.

The patient arrived intubated and sedated with C-collar in place. There were no spontaneous movements of his extremities or withdrawal response from painful stimulus secondary to sedation, but he maintained hemodynamic stability.  Figure 2 illustrates his significant facial trauma.

The patient was noted to have an 18 cm anterior neck wound with degloving down to the carotid artery. Gross contamination and glass were evident in the wound but no significant active bleeding. Appropriate antibiotic and tetanus prophylaxis were administered. Physical examination of his spine with log roll and palpation did not identify stepoffs or deformity of the patient's spine. His posterior neck displayed significant swelling. His rectal tone was intact but diminished. Bulbocavernosus reflex was intact.

**Figure 3:**
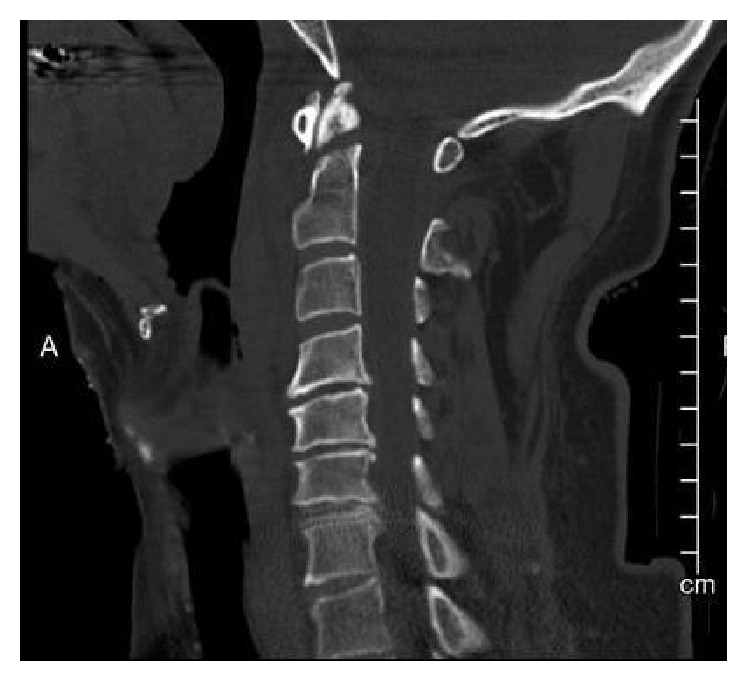
Midaxial sagittal cervical spine CT showing a slightly distracted Type II dens fracture without additional cervical spine fracture.

**Figure 4:**
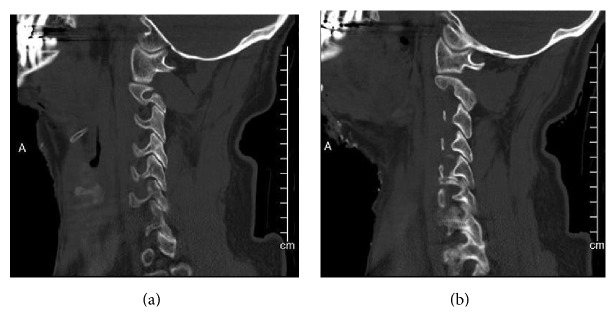
Right and left parasagittal cuts through the occipital-cervical junction demonstrating reduced occipital condyle-C1 and C1-C2 joints bilaterally. There is no evidence of distraction across these joints.

**Figure 5:**
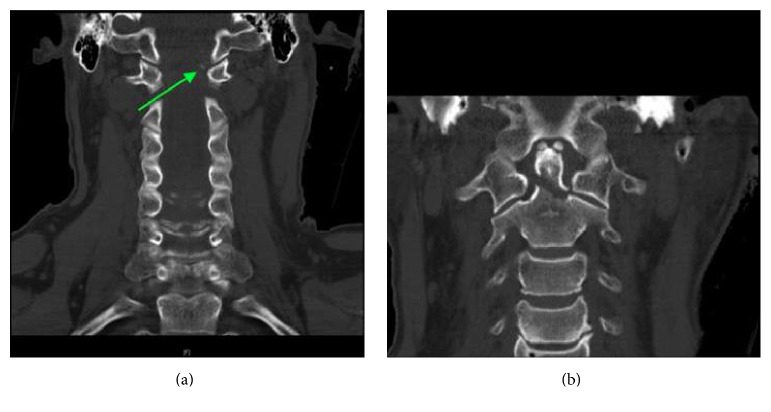
Coronal reconstruction of the cervical spine again demonstrating the reduced occipital condyle-C1 joints bilaterally. There is an avulsion injury of the left occipital condyle consistent with a Type III fracture. The second coronal reconstruction illustrates reduced C1-C2 joint.

The patient's cervical spine CT from the outside facility demonstrated a moderately distracted but relatively well-aligned Type 2 dens fracture (Figure 3). There was no evidence of obvious distraction or translation at either the occipital-C1 junction or the C1-C2 joints on either the sagittal or coronal reformats (Figures 4 and 5). Initial review revealed no other injury, but several subtle findings suggested a more severe injury.

**Figure 6:**
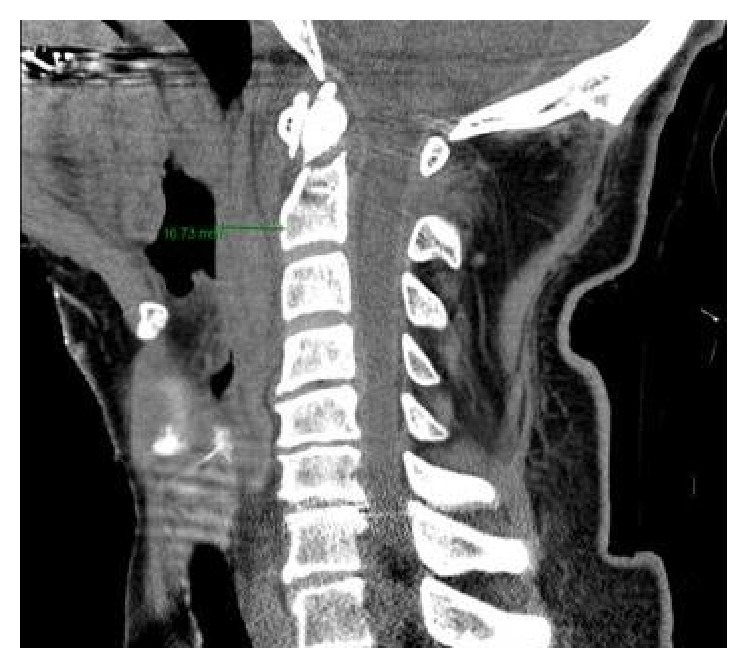
Midsagittal CT soft tissue window demonstrating prevertebral soft tissue swelling of 16.7 mm which is excessive for a transverse Type 2 dens fracture.

**Figure 7:**
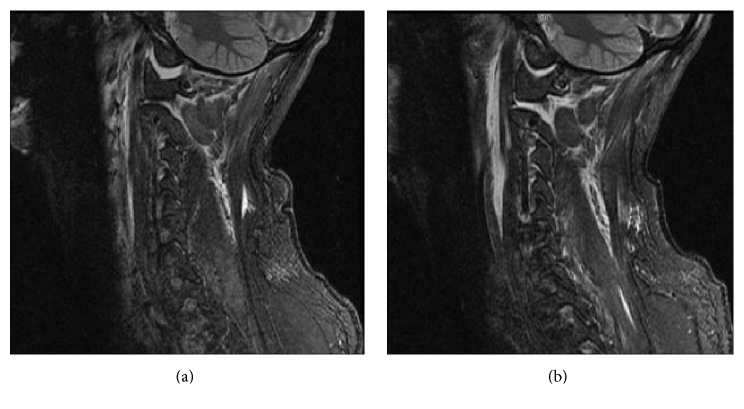
Left and right parasagittal STIR weighted MRI showing displaced occiput-C1 joints.

**Figure 8:**
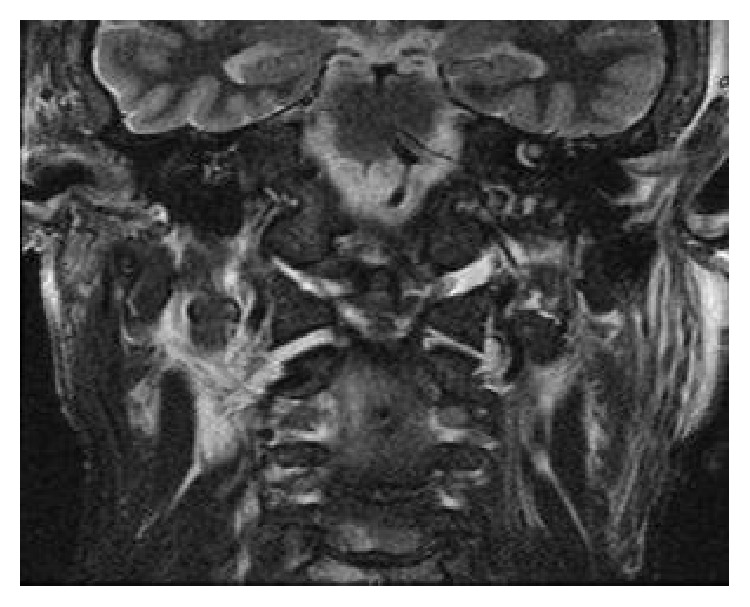
Coronal T2 weighted MRI showing occiput-C1 displacement, C1-C2 displacement, and soft tissue edema in the pericervical spine region.

Given the soft tissue swelling measuring 16.7 mm at C2 on CT scan, the Type III occipital condyle, and the distractive Type II dens fracture, concern for a more substantive injury is raised (Figure 6). Given the above findings, a cervical MRI was obtained several hours later (Figures 7 and 8) to evaluate for craniocervical dislocation. Of note, the cervical collar was on the patient during his MRI as it was for his CT scan.

The cervical MRI demonstrated occipital condyle-C1 subluxation with distraction of the occiput from C1 and C1 from C2 on both the parasagittal and coronal views. Additionally, soft tissue edema at the C1-occiput region indicated a more severe craniocervical injury than initially appreciated. These findings revealed the extent of injury to the craniocervical junction and the highly unstable occipitocervical dissociation. Of significance as well, there was no evidence of brainstem or spinal cord injury on the MRI.

The patient's cervical collar was removed immediately and the patient was placed in bolsters next to his head to try to minimize any distractive forces while optimizing stability prior to definitive fixation. The medical staff was all informed to minimize transfers and mobilization of the patient as much as possible until after operative stabilization.

Given the grave concern regarding the patient's occipitocervical stability and the life-threatening implications with instability at this level, emergent surgery was undertaken. After obtaining baseline electrodiagnostic signals, Mayfield tongs were applied and the patient was transferred to the Jackson table and rotated into a prone position. A lateral fluoroscopic image of the cervical spine verified acceptable alignment of the occipitocervical junction. Electrodiagnostic signals remained stable after positioning with no obvious deficits to SSEP or MEPs.

A midline posterior cervical incision was performed to achieve a subperiosteal exposure of the occiput to the level of C2. Extensive soft tissue disruption and a high degree of instability between the occiput and C2 levels were noted. Patient respirations resulted in changes in alignment between the occiput and C2.

The reduction was confirmed fluoroscopically and instrumentation was placed under fluoroscopic guidance. A left-sided C2 pedicle screw and right-sided C2 laminar screw were selected because of his vertebral artery anatomy. C1 lateral mass screws and an occipital plate were placed. The occipitocervical rods were precontoured to the desired occipital-cervical alignment and used as a reduction aid.

**Figure 9:**
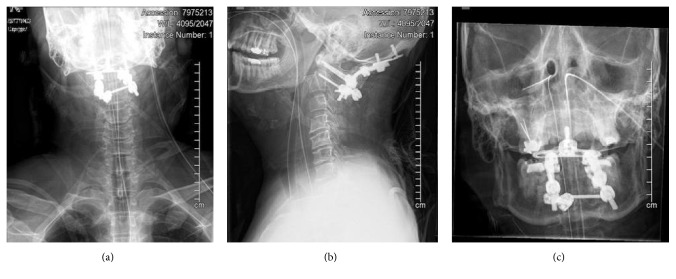
Postoperative imaging demonstrating a reduced and secured occiput to C2 posterior fusion.

The posterior arthrodesis was completed with a combination of morselized locally harvested autologous bone graft, synthetic allograft, and fresh frozen iliac tricortical allograft. The tricortical graft was contoured around the posterior elements between the occiput and C2 and secured with #2 FiberWire wrapped around both rods (Figure 9).

After closure, his care was then turned over to the Otolaryngology Service for neck exploration and complex wound closure.

Upon extubation, he was found to have no neurological deficits. He was discharged home ten days after presentation with no neurologic sequelae.

## 3. Discussion

Craniocervical dissociation is a severe injury that, untreated, can result in significant neurological deterioration and even death. There are many challenges associated with the diagnosis of injuries to the craniocervical junction, not the least of which is the elaborate osseoligamentous anatomy and its complicated radiographic projection. Diagnostic challenges were highlighted by Bellabarba et al. who reported an average delay of diagnosis of 2 days in 13/17 patients (76%), with 5 of these recognized only after the delayed onset of neurological symptoms [6].

Initial emergency department imaging protocols for the evaluation of the cervical spine commonly include supine plain films and fine-cut CT scans with coronal and sagittal reconstructions. Traditional evaluation of the CCJ on these studies relies heavily on Harris lines [8]. Harris lines were originally utilized in plain lateral radiographs but are currently widely extrapolated by radiologists to use in sagittal reformats on CT scans. Clearly, there are technical differences related to magnification issues amongst other things, yet Harris lines are still a major go-to in assessing normal craniocervical parameters. The basion-dens interval (BDI, distance from basion to upper odontoid tip) should be <12 mm in 95% of patients. The basion-axis interval (BAI, distance between line projecting cranially from the posterior cortex of C2 body and the basion) should also be <12 mm in 98% of the population [8]. While retrospective review of initial injury radiographs in patients with CCD found abnormal measurements in 94% of cases, these measures do not capture all cases of instability [6]. Deviations from these known radiographic norms and the presence of other clinical indicators should prompt further evaluation of the CCJ. The case presented here, however, presented with normal Harris measurements on injury plain films and CT scans.

Certainly, with the increasing use of CT scans as a “first” modality and less use of plain radiographs, other measurements besides Harris lines have also been described to aid in identifying CCDs. Chaput et al. have recommended an assessment on CT scan of the lateral mass interval (LMI) within C1-C2 with the normal defined as less than 4 mm and using a BDI of less than 10 mm instead of 12 mm as reliable assessors of cranial cervical injuries [9]. Radcliff et al. looked at 42 different anatomical measurements and concluded that direct measurements of the joint space had the least variation with a mean OC joint space of 0.6 mm in sagittal and coronal measurements with an upper 95% confidence interval (CI) of 1 mm. The mean AA joint space was 0.6 mm, with an upper 95% CI of 1.2 mm at the lateral aspect of the joint on the coronal image only [10].

As highlighted by this case, patients with high-energy injuries or significant trauma to the upper neck and head are susceptible to injuries to the CCJ. Chaput et al. reported 16 cases of CCD after high-energy mechanisms and 6 deaths with high cervical spinal cord transections. In addition to abnormal Harris measurements, there are other clues to the diagnosis of CCD. Distracted Type II dens fractures in high-energy trauma patients are one such indication. This abnormal fracture pattern results from a distraction displacement force vector. Additionally, Type III occipital condyle fractures are known to result from distractive injuries to the CCJ and their presence should prompt further investigation to insure no further injury to the CCJ is present. The combination of these two injuries in the setting of high-energy trauma and significant soft tissue trauma to the head/neck area are highly suspicious for injury to the CCJ. Even with reduced Occiput-C1 and C1-C2 articulations, further investigation into CCJ stability is warranted.

Some patients sustain CCD, with or without fracture, but their CCJ osseous articulations reduce spontaneously and symmetrically. In these cases, as presented here, the extent of injury and instability at the CCJ may be underappreciated and clinical clues are essential to raise suspicion of CCJ injury. MRI is a powerful tool in this setting. It can provide important information about soft tissues injuries of the occipital-cervical junction that may not be appreciated on plain radiographs or CT scans. Additionally, stability of the CCJ is not always clear.

In cases of CCD with reduced osseous articulations and advanced imaging indicating soft tissue injury, the need for surgical intervention may remain unclear. Dynamic traction fluoroscopy can help the treating surgeon understand the extent of instability at the CCJ in this setting. Child et al. evaluated cadaveric specimens and found CCD to be an “all or nothing” phenomenon [3]. That is, either the CCJ is stable or it is not and dynamic traction fluoroscopy can clarify this when advanced imaging studies are inconclusive. While this practice has limited indications in the initial evaluation of trauma patients, it can provide valuable information regarding the need for surgical intervention in unclear clinical settings and can be performed safely in the hands of a competent spine surgeon.

After diagnosis of CCD is made, to lessen the risk of further injury, patients with CCD should have the CCJ immobilized and definitively stabilized emergently. Traction is strictly contraindicated and cervical collars should be avoided as they can produce longitudinal distraction [11].

Surgical stabilization of the CCJ requires at least occiput to C2 rigid posterior cervical instrumentation and fusion [6]. Many of the ligamentous stabilizers of the CCJ extend caudally to the C1 ring. For this reason, stopping fixation constructs short of C2 would be ineffective, even if the primary distractive injury were identified between the occiput and C1. Occiput to C2 fusion for this injury is a reliable technique with 0/48 patients receiving fusion for CCD developing pseudarthrosis or hardware failure [7].

## 4. Conclusion

While CCD is generally a neurologically devastating injury, a high index of suspicion, even without abnormal traditional radiographic measurements, may prevent injury in select patients who present without gross cord compromise. Careful consideration of associated fractures, soft tissue injuries, and mechanism of injury are essential clues to the correct diagnosis and management of injuries to the CCJ.
